# Improving the Prediction of Prostate Cancer Overall Survival by Supplementing Readily Available Clinical Data with Gene Expression Levels of IGFBP3 and F3 in Formalin-Fixed Paraffin Embedded Core Needle Biopsy Material

**DOI:** 10.1371/journal.pone.0145545

**Published:** 2016-01-05

**Authors:** Zhuochun Peng, Karl Andersson, Johan Lindholm, Olga Dethlefsen, Setia Pramana, Yudi Pawitan, Monica Nistér, Sten Nilsson, Chunde Li

**Affiliations:** 1 Department of Oncology-Pathology, Karolinska Institutet, Stockholm, Sweden; 2 Department of Immunology, Genetics and Pathology, Uppsala University, Uppsala, Sweden; 3 Clinical Pathology/Cytology, Karolinska University Hospital, Stockholm, Sweden; 4 Department of Medical Epidemiology and Biostatistics, Karolinska Institutet, Stockholm, Sweden; 5 Clinical Oncology, Karolinska University Hospital, Stockholm, Sweden; 6 Chundsell Medicals AB, Stockholm, Sweden; 7 Ridgeview Instruments AB, Uppsala, Sweden; The Chinese University of Hong Kong, HONG KONG

## Abstract

**Background:**

A previously reported expression signature of three genes (IGFBP3, F3 and VGLL3) was shown to have potential prognostic value in estimating overall and cancer-specific survivals at diagnosis of prostate cancer in a pilot cohort study using freshly frozen Fine Needle Aspiration (FNA) samples.

**Methods:**

We carried out a new cohort study with 241 prostate cancer patients diagnosed from 2004–2007 with a follow-up exceeding 6 years in order to verify the prognostic value of gene expression signature in formalin fixed paraffin embedded (FFPE) prostate core needle biopsy tissue samples. The cohort consisted of four patient groups with different survival times and death causes. A four multiplex one-step RT-qPCR test kit, designed and optimized for measuring the expression signature in FFPE core needle biopsy samples, was used. In archive FFPE biopsy samples the expression differences of two genes (IGFBP3 and F3) were measured. The survival time predictions using the current clinical parameters only, such as age at diagnosis, Gleason score, PSA value and tumor stage, and clinical parameters supplemented with the expression levels of IGFBP3 and F3, were compared.

**Results:**

When combined with currently used clinical parameters, the gene expression levels of IGFBP3 and F3 are improving the prediction of survival time as compared to using clinical parameters alone.

**Conclusion:**

The assessment of IGFBP3 and F3 gene expression levels in FFPE prostate cancer tissue would provide an improved survival prediction for prostate cancer patients at the time of diagnosis.

## Introduction

The last two decades have brought considerable advances in the understanding of the molecular abnormalities that are associated with cancer prognosis. Accurate classification of cancer is of great practical value in the clinical management of patients. In particular, the use of genetic information and gene expression assays as an aid in cancer prognosis assessments is increasing [1]. The scientific community is approaching consensus in that comprehensive molecular characterization of critical elements of cancer disease, such as gene expression, will be key for developing new successful prognostic assays [2].

In a previous study from our laboratory the measurement of a gene signature expression levels in fresh frozen Fine Needle Aspiration (FNA) cytology samples was shown capable of estimating the overall survival time at diagnosis for prostate cancer patients [3]. The gene signature provided additional prediction power in terms of patients’ survival compared to the clinical parameters, such as age at diagnosis, cytology WHO grade, tumor stage and PSA value. Gleason score (GS) cannot be determined for FNA samples.

Currently, one of the most common sample types in clinical practice for prostate cancer diagnosis is the formalin fixed paraffin embedded (FFPE) core needle biopsy, which can be used for Gleason grading by pathologists. Age at diagnosis is an important risk factor for prostate cancer patients, which is also believed to be a dominant prognostic parameter for predicting overall survival [4]. GS has also been one of the standard prognostic parameters for estimating the aggressiveness of prostate cancer for decades [5]. In order to investigate the relations of gene expression levels and GS together with age at diagnosis of patients, we conducted a new cohort study using FFPE tissue samples with two control alive groups: one group where GS and age at diagnosis were matched and one randomly selected.

An advantage of FFPE samples is that they can be easily archived and that many cohorts have long time follow-up clinical data available, which greatly facilitates clinical studies. Even though the extracted RNA from FFPE samples may be of relatively low quality, multiple recent studies have shown promising results when utilizing degraded RNA extracted from archival FFPE samples for quantifying gene expression levels by optimized RT-qPCR methods [6–8]. One example is the Prostatype RT-qPCR kit, which is developed and optimized for measuring the gene expression levels of a gene signature of IGFBP3, F3 and VGLL3 particularly in FFPE samples [9].

One important factor when analyzing patient material is to minimize operator dependencies. A recent study reported that the operator dependent choice of FFPE core needle biopsy (with different Gleason patterns) for measurement of expression levels of IGFBP3 and F3 has limited impact on the results, when using the Prostatype RT-qPCR kit [9]. The effect on VGLL3 measurements could not be estimated in that study due to limited tissue input.

In this work, we have evaluated archived FFPE core needle biopsy samples taken from a cohort of 241 patients who were diagnosed from 2004 to 2007 in the Stockholm region, Sweden. We only analyzed the gene expression levels of IGFBP3 and F3, measured only on primary Gleason pattern tissue samples. There were two groups of deceased patients, with prostate cancer death within 5 years and death due to other diseases within 5 years. We used two control groups, one matched alive group where GS and age were matched to the deceased groups, and one randomly selected alive patient group. The main purpose of the study was to determine whether there were any differences in expression levels of IGFBP3 and F3 within these different patient groups with significantly different survival time. We also attempted to verify whether the gene signature in archive FFPE core needle tissue samples combined together with current clinical parameters can provide better prediction accuracy in terms of patients’ survival time, compared to the prediction made by the clinical parameters only.

## Materials and Methods

### Ethics Statement

The study protocol was approved by the Research Ethics Committee of the Karolinska Institutet (approval number: 00–164) and was performed in accordance with the ethical principles described in the 1964 Declaration of Helsinki.

Informed written consent for general bio-bank material collection was obtained from all participants according to the Swedish bio-bank law, prior to their inclusion in the study.

### Clinical Characteristics of the Cohort and Sample Type

A population based cohort of 2053 prostate cancer patients diagnosed from 2004 to 2007, were extracted from the cancer patients registry data in the Regional Cancer Centrum Stockholm Gotland, Sweden. From this registry cohort, all patients that had died of prostate cancer before the end point of follow-up time (the end of 2013) were selected, together with other types of patients, composed into a study cohort of 241 prostate cancer patients (Table 1). The study cohort was designed by first defining 4 groups: patients who died of prostate cancer within 5 years (PCa group), patients who died of other diseases (OD group) within 5 years, patients who survived at least 5 years selected by matching the PCa and OD groups with the distribution of age at diagnosis and Gleason Scores (MA group), and randomly selected patients who survived at least 5 years (RA group). Each group initially consisted of 80–85 patients, with similar number of patients diagnosed from each year. The matched alive patients (MA patients) were aiming to be proportionally selected by matching their ages at diagnosis and Gleason scores to those of patients who were deceased (PCa and OD patients), while the other alive patients (RA patients) were randomly selected. When retrieving archival samples for all patients, some tissue samples were missing, some were clearly damaged, and some did not have sufficient biopsy material to allow measurements. Data was collected on 64 patients in the PCa group, 60 in the OD group, 63 in the MA group, and 54 in the RA group.

**Table 1:** Characteristics of the patients.

	PCa	OD	MA	RA	Total
Patients, n (%)	64 (26.6)	60 (24.9)	63 (26.1)	54 (22.4)	241 (100.0)
Survival, years: Median (range)	2.6 (0.2–6.6)	2.1 (0.1–6.6)	7.7 (5.7–9.5)	7.5 (5.7–9.4)	5.7 (0.1–9.5)
Age, years: Mean±SD	73.2±9.3	76.0±8.2	71.0±9.2	67.1±7.8	71.9±9.2
Diagnosis year: n(%)					
2004	13 (20.3)	14 (23.3)	14 (22.2)	11 (20.4)	52 (21.6)
2005	17 (26.6)	16 (26.7)	20 (31.7)	14 (25.9)	67 (27.8)
2006	15 (23.4)	17 (28.3)	13 (20.6)	10 (18.5)	55 (22.8)
2007	19 (29.7)	13 (21.7)	16 (25.4)	19 (35.2)	67 (27.8)
PSA, ng/ml^a^: n (%)					
≤ 10	11 (18.0)	27 (46.6)	33 (52.4)	35 (64.8)	106 (44.9)
> 10 and ≤ 20	12 (19.7)	9 (15.5)	11 (17.5)	9 (16.7)	41 (17.4)
> 20 and ≤ 50	14 (23.0)	18 (31.0)	16 (25.4)	7 (13.0)	55 (23.3)
> 50	24 (39.3)	4 (6.9)	3 (4.8)	3 (5.6)	34 (14.4)
Missing	3	2	0	0	5
Gleason Score					
≤ 6	3 (4.8)	20 (33.9)	17 (27.9)	23 (43.4)	63 (26.8)
7	20 (32.3)	24 (40.7)	24 (39.3)	24 (45.3)	92 (39.1)
≥ 8	39 (62.9)	15 (25.4)	20 (32.8)	6 (11.3)	80 (34.0)
Missing	2	1	2	1	6
Clinical stage: n (%)					
T1c	2 (3.2)	25 (42.4)	27 (42.9)	22 (41.5)	76 (31.9)
T2	17 (27.0)	16 (27.1)	19 (30.2)	22 (41.5)	74 (31.1)
T3/T4/N1/M1^b^	44 (69.8)	16 (30.5)	16 (27.0)	9 (17.0)	88 (37.0)
Missing	1	1	0	1	3
Treatment: n (%)					
Prostatectomy	1 (1.6)	4 (6.7)	15 (23.8)	18 (33.3)	38 (15.8)
Radiation	3 (4.7)	2 (3.3)	9 (14.3)	9 (16.7)	23 (9.5)
Hormone	48 (75.0)	22 (36.7)	15 (23.8)	3 (5.6)	88 (36.5)
Missing	12 (18.8)	32 (53.3)	24 (38.1)	24 (44.4)	92 (38.2)

Abbreviations: PCa: Death due to prostate cancer; OD: Death due to other causes; MA: Matched Alive more than 5 years; RA: Randomized Alive more than 5 years.

^a^ PSA levels in serum were measured at the time of diagnosis (before treatment).

^b^ Patient’s clinical stage is either T3 or T4 or N1 or M1.

The FFPE prostate core needle biopsy samples were collected at the time of prostate cancer diagnosis according to the routine procedure used at Aleris Diagnostik AB (Aleris Medilab) in Stockholm, Sweden. For each patient, 6–10 core needle biopsies were taken for Gleason grading [10]. Positive biopsies taken from those patients were used for this study. All biopsies had been stored in the Aleris Medilab biobank facility at conditions suitable for FFPE samples for at least 6 years before use in this analysis.

### Sample Preparation and RNA Extraction

The sample preparation process is described by Peng et al [9]. The results reported here were mainly obtained at the same time as the results reported by Peng et al [9]. In brief, prior to RNA extraction, the FFPE tissue sample preparation process is composed of three steps: (a) Sectioning of FFPE core needle biopsy blocks; (b) Marking and quantifying cancer area for scraping; (c) Scraping cancer cell-containing area for the sample measurements.

#### Sample measurements

We previously reported that the gene expression levels of IGFBP3 and F3 in prostate cancer epithelial cell-containing tissue representing the primary and secondary Gleason patterns were high and consistent [9]. In the present study we analyze IGFBP3 and F3 gene expression levels only from the sample measurements with respect to the primary Gleason tumor pattern. A pathologist collected tissue from cancer areas with the primary Gleason pattern type of cells from one or more biopsies to harvest sufficient tissue for analytical procedure, although it was later proven that VGLL3 expression measurements required more tissue input [9]. The percentage of tumor material in each cancer cell-containing sample was evaluated and confirmed by pathologists using digitally scanned images of H&E stained slides. More than 93% of sample measurements contained >70% cancer epithelial cells. The details of sample measurement procedures were also described in our previous study [9].

#### Total RNA extraction

Total RNA was extracted from scraped tissue samples using the High Pure FFPE RNA Micro Kit (Roche Applied Science/ Roche Diagnostics GmbH, catalog number: 4823125001) according to vendor´s instruction. Extracted RNA was immediately subjected to the RT-qPCR analysis.

### One-Step RT-qPCR Reaction

Expression levels of IGFBP3, F3, VGLL3 and GAPDH were measured using a pre-production version of the commercial Prostatype RT-qPCR kit, (Chundsell Medicals AB, Sweden). This is a four-multiplex one-step RT-qPCR kit, which is designed to measure gene expression levels of the three biomarker genes IGFBP3 (insulin-like growth factor binding protein 3), F3 (coagulation factor III (thromboplastin, tissue factor)), and VGLL3 (vestigial-like family member 3), normalized to the expression level of the gene GAPDH (glyceraldehyde 3-phosphate dehydrogenase) in RNA extracted from FFPE human prostate cancer tissue samples. The sequence information of probes and primers has been reported previously [3]. According to the instructions for use (IFU), extracted total RNA can be used for this RT-qPCR reaction immediately even without quantifying the input of RNA. The instructions for use also recommend to exclude those sample measurements with Ct (GAPDH) > 29.0, possibly due to too low amount of extracted RNA. In total 50 patient samples (17.2% dropout rate) out of 291 patient samples were excluded due to this reason. The kit further contains positive and negative controls, which were assayed together with each batch of prostate cancer tissue samples. Measurements were conducted using Roche LightCycler 480 instrument II, a qPCR platform on which a color compensation method was run prior to performing the qPCR analysis.

### Data Analysis

Ct values of all batches of samples were extracted according to instructions from the manufacturers (Roche and Chundsell Medicals). A batch of RT-qPCR experiments was considered valid only if positive and negative controls were valid. Samples with GAPDH Ct value of > 29.0 were excluded according to the Prostatype RT-qPCR kit IFU. The expression levels of the IGFBP3 and F3 genes were normalized to that of GAPDH and were presented as the delta Ct value, which is inversely correlated to the gene expression level. For VGLL3, the expression data was not included for analysis due to inadequate tissue input reported in our previous publication [9]. These delta Ct values were plotted versus Gleason Score for each patient belonging to groups RA, OD and PC and were used for the further statistical analyses such as the TTEST and the kNN modeling. A drawback of the use of multiple matched patient groups is that conventional survival analyses such as Kaplan Meier survival analysis or Cox proportional analysis would suffer from statistical bias.

#### Student’s t-test (TTEST) analyses

With an assumption of equal two-tailed distribution variances, two-sample Student’s *t*-tests within different groups were performed such as ‘MA group vs. PCa group’, ‘MA group vs. OD group’. etc. (Table 2). All variables are modeled as continuous variables, such as age at diagnosis, Gleason score, tumor stage, log10 (PSA value), delta Ct IGFBP3 and delta Ct F3.

**Table 2:** TTEST analyses within groups.

	Delta Ct IGFBP3	Delta Ct F3	log(PSA)^a^	Age, year	Gleason score	Tumor stage
RA						
Mean	3.46	1.55	1.00	67.07	6.74	1.77
STD	1.94	1.90	0.37	7.75	0.90	0.78
N	49	49	54	54	53	53
TTEST^b^, *P*						
RA vs. PCa	0.5271	0.0026*	<0.0001*	0.0002*	<0.0001*	<0.0001*
RA vs. OD	0.0044*	0.0255*	0.0290*	<0.0001*	0.1633	0.7100
RA vs. MA	0.0008*	0.0280*	0.1451	0.0150*	0.0158*	0.8100
MA						
Mean	4.74	2.59	1.12	70.98	7.21	1.81
STD	1.86	2.76	0.46	9.16	1.14	0.82
N	57	57	63	63	61	63
TTEST^b^, *P*						
MA vs. PCa	<0.0001*	0.2786	<0.0001*	0.1750	<0.0001*	<0.0001*
MA vs. OD	0.8033	0.9186	0.5972	0.0018*	0.2346	0.8888
OD						
Mean	4.65	2.54	1.16	76.01	6.98	1.83
STD	2.18	2.48	0.37	8.23	0.96	0.83
N	55	55	58	60	59	59
TTEST^b^, *P*						
OD vs. PCa	0.0002*	0.2290	0.0001*	0.0794	<0.0001*	<0.0001*
PCa						
Mean	3.24	3.21	1.58	73.21	8.10	2.75
STD	1.77	3.35	0.69	9.28	1.07	0.72
N	61	61	61	64	62	63
TTEST^b^, *P*						
MA vs. [OD+PCa]	0.0113*	0.5234	0.0031*	0.0100*	0.0636	0.0004*

Abbreviations: PCa: Death due to prostate cancer; OD: Death due to other causes; MA: Matched alive patients lived more than 5 years; RA: Randomly selected alive patients lived more than 5 years.

^a^ PSA levels in serum were measured at the time of diagnosis (before treatment).

^b^ TTEST analyses have been performed in two-tailed. *P* values are calculated within two groups, if *P*<0.05, this is marked with a star.

#### k Nearest Neighbor (kNN) modeling

Out of 241 patient samples, 152 had complete data for both gene expression and clinical variables. The data set was randomly divided into a training set (68% of the data set, n = 107) for model development and a test set (32% of the data set, n = 45) for model verification. Two different kNN models estimating overall survival were designed and optimized on the training set data (Table 3). One of the models had only clinical parameters, and one had clinical parameters combined with the gene expression values of IGFBP3 and F3. Euclidian distance measures were used and the average survival time for the two nearest neighbors was calculated as output. Firstly, the training set was used to obtain the scaling weight for parameters to zero average and unit variance, which was applied to both the training set and the validation set. The weight of each parameter was determined from the training set, first through an exhaustive search of all combinations of the weight factors 1 and 3, then through testing 100000 times randomly with 10% variations of the weight factors. The identified weights of parameters derived from the training set were finally applied on the test set. For both models, the prediction performance of the kNN models was evaluated by comparing the average absolute prediction error. Survival time longer than 6 years was treated as exactly 6 years survival. The kNN method is a pattern based classification tool that assigns an unknown case to the same group as the most similar reference cases, meaning that kNN is capable of classifying data sets where there is no simple univariate relationship between gene expression levels and patient outcome.

**Table 3:** Analysis of kNN performance (Classification error).

Model	Training set, N = 107(68%)	Validation set, N = 45(32%)	*P*^b^
	Average error^a^, year	Average error^a^, year	
Clinical parameters^c^	1.97	2.13	0.0040*
Clinical parameters +Genes^d^	1.42	1.94	

^a^ Average error is the average absolute prediction error.

^b^ *P* value is generated by a two-tailed TTEST based on the average survival time prediction errors derived from two models. If *P*<0.05, a star is marked.

^c^ Clinical parameters are composed of Age at diagnosis, Gleason Score (GS), log10(PSA value) and Tumor Stage, their scale weight in both models are [Age, GS, logPSA, Tumor Stage] = [1.245, 4.326, 0.340, 1.129]. Gleason score is modeled as a continuous variable. Tumor stage is modeled as a continuous variable.

^d^ Gene variables are composed of gene expression levels of IGFBP3 and F3, presented as Delta Ct values of genes (delta Ct IGFBP3, delta Ct F3). Their scale weight in modeling is [IGFBP3, F3] = [0.923, 1.017].

#### Multiple linear regression analysis

The expression levels of genes, delta Ct IGFBP3 and delta Ct F3, were fit in the multiple linear regression models in order to investigate whether the association between ‘gene variable’ and ‘grouping variable’ was related or influenced by the other variables such as age variable and Gleason score variable (S1 Table). MA group and RA group were separately integrated with PCa and OD groups to compose two major types of models: Type 1: MA, OD and PCa groups; Type 2, RA, OD and PCa groups.

#### Nominal logistic regression modeling

Four groups of patients were modeled as a nominal variable as ‘grouping variable’; the prediction of grouping variable was modeled using a multi-nominal logistic regression model. We fit the multi-nominal logistic regression models between the ‘grouping variable’, either with four clinical parameters only (Age, log (PSA), Gleason score and tumor stage), or with four clinical parameters combined together with gene variable (the expression levels of IGFBP3 and F3). Gleason score and tumor stage are modeled as category variables with three categories respectively: Gleason score ≤6, 7 or ≥8; tumor stage = T1c, T2 or T3/T4/N1/M1. Receiver Operating Characteristic (ROC) curves were generated using JMP^®^ statistics software (version 8.0.1, SAS Institute, Inc.) for each subgroup prediction showing the sensitivity and the specificity of grouping prediction.

## Results

241 patients, diagnosed from 2004 to 2007 with archive FFPE core needle biopsies of sufficient quantity and quality, were analyzed in this study. The survival time of alive patients was calculated with respect to the end-point of follow-up (end of 2013). The median survival time was 5.7 years with a range of 0.1 to 9.5 years (Table 1). The cohort was composed of two main groups: deceased groups and alive groups, with similar number of patients in each main group. For deceased groups, there were the prostate cancer-specific deceased group (PCa group), and the deceased group who died of other diseases (OD group) with short median survival time 2.6 and 2.1 years respectively. The matched alive group (MA group) and the alive group randomly selected (RA group) were two alive groups with longer median survival time 7.7 and 7.5 years respectively. The MA patients had higher ages (71.0 years) at diagnosis as compared to RA patients (67.1 years) with P value 0.0150; the two groups with deceased patients also had higher ages at the time of diagnosis (PCa, 73.2 years; OD, 76.0 years).

In parallel with the sample measurements, the positive control sample was measured 49 times. The positive control sample contains four synthetic RNA plasmid copies integrated with their respective inserts of IGFBP3, F3, VGLL3 and GAPDH cDNA amplicons, which can be amplified into pre-designed Ct values as the positive control according to IFU of RT-qPCR kit. For IGFBP3 positive control measurements, the reported delta Ct value was in average 3.65, the standard deviation was 0.37, and the max/min values were 4.44/2.62. For F3, the corresponding values were 1.90; 0.32; 2.65/0.71, and for VGLL3, they were 5.13; 0.36; 5.91/3.76. Based on this, the typical variation of delta Ct values from run to run due to the assay as such was estimated to be two standard deviations, i.e. 0.73 for IGFBP3, 0.64 for F3 and 0.73 for VGLL3. This is in line with previously reported values [9], indicating that the measurements of qPCR runs were valid.

For gene expression levels of IGFBP3 and F3, there were significant differences within different groups (Table 2). IGFBP3 was expressed at lower levels in the MA patient group (mean delta Ct 4.74± 1.86) as compared to PCa group (mean delta Ct 3.24±1.77) with P<0.0001. The PCa group also had higher expression levels of IGFBP3 (P = 0.0002) as compared to the OD patient group. Although there was not significant expression difference between the OD group (mean delta Ct 4.65± 2.18) and the MA group, IGFBP3 expression levels could significantly stratify between the MA group and the combined group of deceased patients (PCa+OD) with *P* value 0.0113. At the same time we also observed Gleason score values were not significantly different between the MA group and the combined PCa+OD group, indicating that matching procedure was adequate. This indicated that the possibility to stratify deceased patients with matched alive patients using the expression level of IGFBP3 was most likely related to the gene expression itself and not to the Gleason core parameter. Diagnosis age was lower in MA group as compared to the combined PCa+OD group with *P* = 0.0100, indicating that the matching conducted at the original data set did not withstand that about 17% of samples were lost for analysis.

To better understand if the diagnosis age or other clinical parameters may have a stratification propensity, we performed the multiple linear regression analyses between the IGFBP3 expression variable associated with the ‘grouping’ variable, age at diagnosis and other clinical parameters (S1 Table). Irrespective of age at diagnosis variable, IGFBP3 expression level still maintained its significance to stratify MA group and PCa group. For patients with GS > = 8, GS had an even more dominant contribution to stratify MA group from deceased groups. F3 had the same contribution to stratify RA group with OD group irrespective of GS.

F3 had a significantly increased expression level in random alive patients, RA group, as compared to any of the other three groups (Table 2). This suggested that F3 expression levels could provide an additional value to stratify the ‘baseline’ group (RA group) with the other three groups after IGFBP3 distinguished MA alive patients from deceased groups.

**Fig 1:**
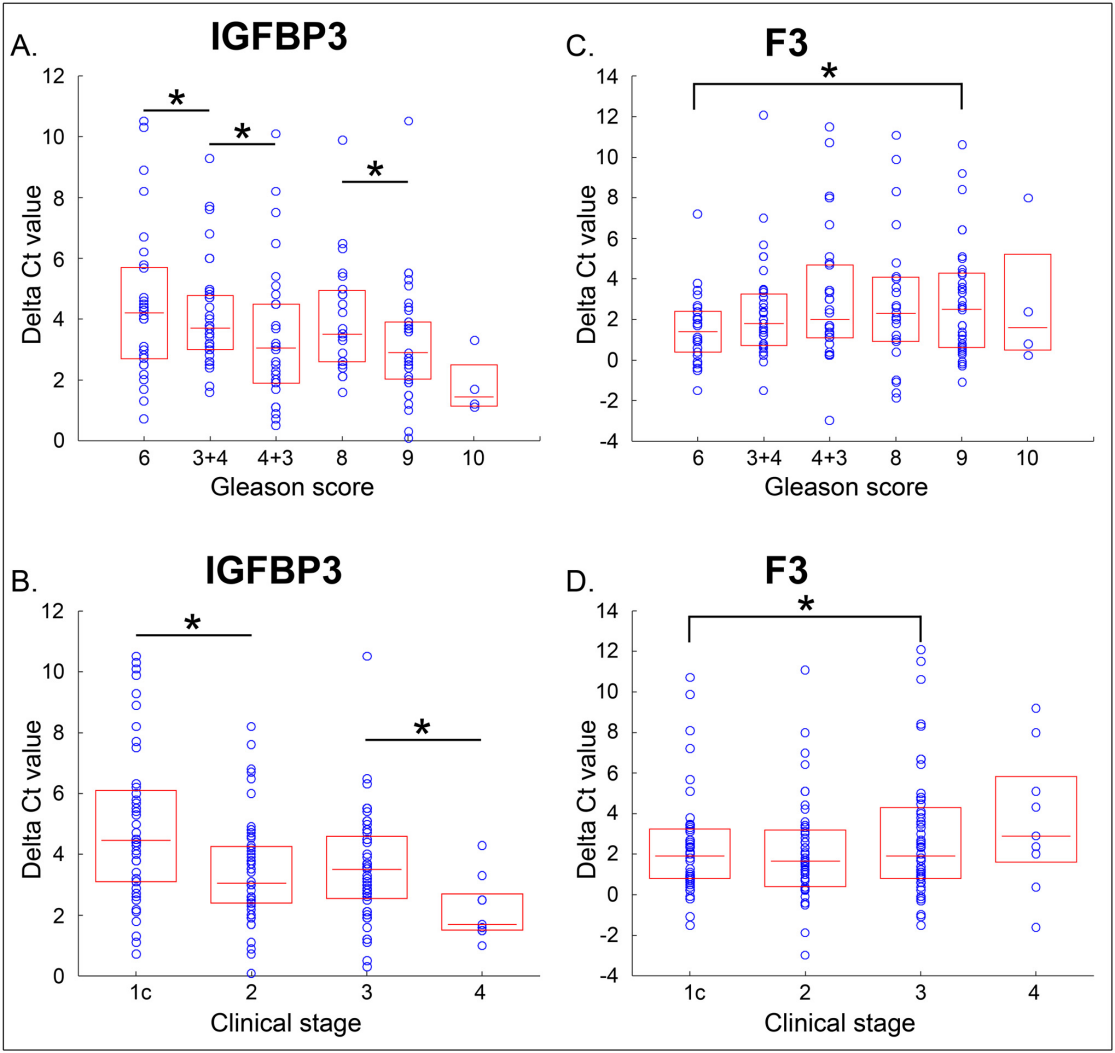
Delta Ct values distribution graphs of IGFBP3 and F3 according to Gleason score and clinical stage. Upper panels (A) and (C), are box plots of Delta Ct values for IGFBP3 and F3 for subcategories of Gleason score value. Each patient is represented as a blue circle. The box covers the two center quartiles, and the median value is represented as a horizontal line in the box. The same data of delta Ct values for the two genes were plotted for each clinical stage subcategory (Lower panels: (B) and (C)). Delta Ct for IGFBP3 decreased with increasing Gleason Score and Delta Ct for F3 increased slightly with increasing Gleason score. The significances of expression level differences between subgroups were analyzed by TTEST, differences between subgroups marked with stars meaning P<0.05.

**Fig 2:**
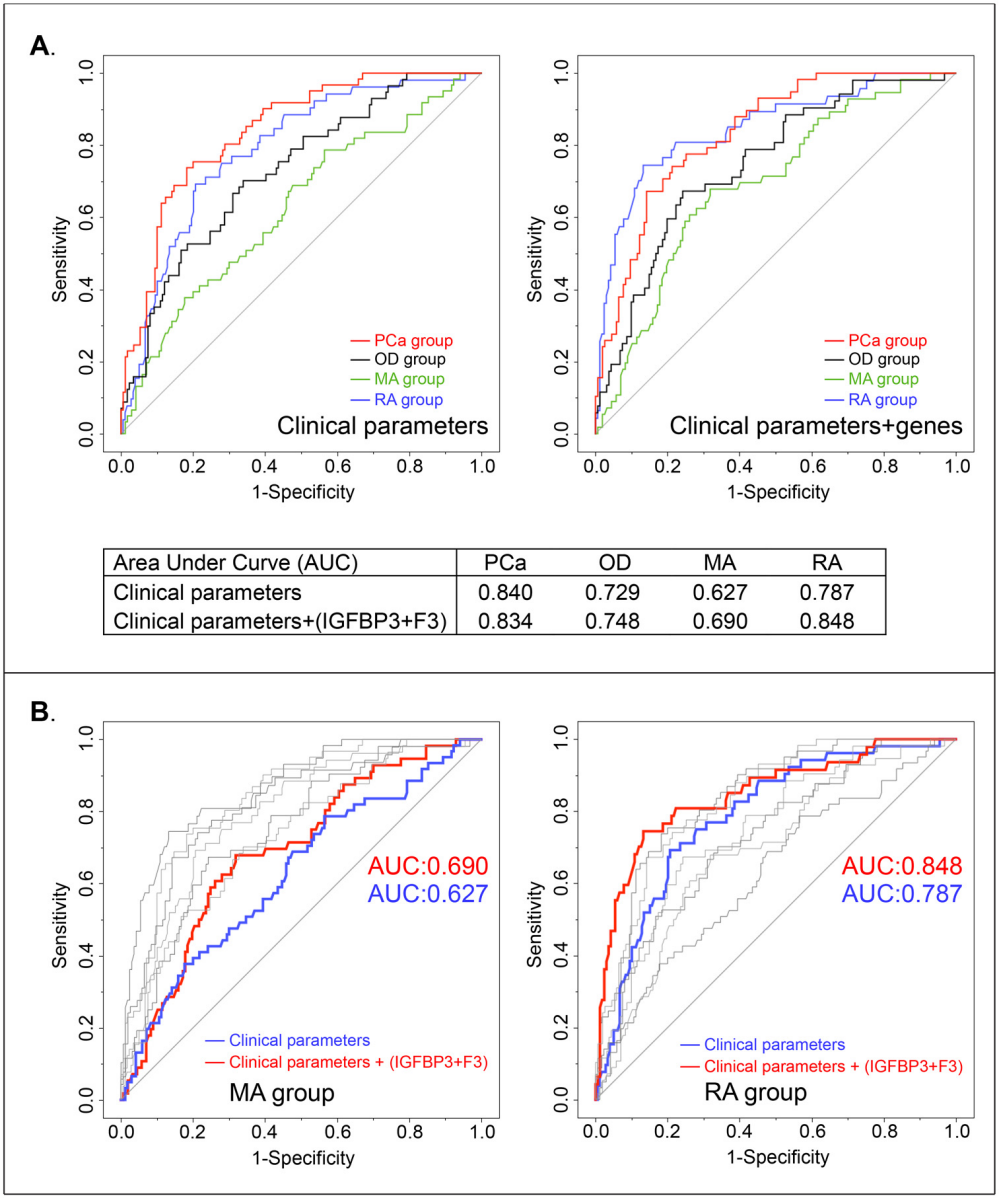
ROC curves for grouping variable survival prediction. (A) The grouping variable predictions for four patient subgroups (RA group: blue line, MA group: green line, OD group: black line and PCa group: red line) were determined by the clinical parameters alone (Left part), and by both clinical parameters and the expression levels of IGFBP3 and F3 (Right part). The area under the curve (AUC) values of predictions for OD, MA and RA groups were increased by adding expression levels of the two genes. (B) We overlapped all ROC curves generated from the two models in one graph, color marked the ROC curves only for MA group (Left part) and RA group (Right part). ROC curves modeled from clinical parameters only are marked with red color, from clinical parameters combined together with the two gene variables are marked with blue color. We observed that adding gene variables increased AUC values of predictions for MA and RA groups.

In order to investigate the gene expression levels of two genes within different ranges of clinical parameters, we plotted Delta Ct values of two genes in relation to different values of GS or clinical parameters. We combined PC, OD and RA groups together since these three groups are representative of the whole population. With in totally 178 patients, we analyzed gene expression levels of IGFBP3 and F3 according to subcategories of Gleason score and clinical stage. The Delta Ct values of two genes within different ranges of GS and clinical stage were plotted as box plots (Fig 1). We observed decreasing delta Ct values for IGFBP3 with increasing GS, and slightly increasing Delta Ct for F3 with increasing GS as seen in Fig 2(A) and 2(C). This means IGFBP3 expression was high in high GS tumors, while F3 expressed low in high GS tumors. We observed a similar pattern when comparing to clinical stage as seen in Fig 1(B) and 1(D).

To assess the survival predictive performance of the genes IGFBP3 and F3, two different kNN classification models were developed using the training set to estimate the overall survival of patients in the validation set [11]. By randomly selecting subjects into a training set and a validation set, independent verification of the model as developed on the training set was made possible. Two kNN models were developed: one using only four clinical parameters (Age, GS, log(PSA) and tumor stage); the second one using four clinical parameters combined together with two gene expression variables. The scale weight of each parameter is shown in Table 3, of which the weights of the four clinical parameters were the same in the two models.

Better performance (prediction average error = 1.42 year) was obtained when both clinical parameters and gene expression was included in the model, as compared to using clinical parameters only (prediction average error = 1.97 year). To confirm this improvement we observed in a training set, the remaining portion of the data set (about 1/3) was used as a validation set for independent verification of the model. The validation set was evaluated by applying the model built on the training set, and we found that the prediction errors on the validation set were smaller when clinical parameters were combined with gene expression (prediction average error = 1.94 years), as compared to clinical parameters alone (prediction average error = 2.13 years). The performance difference of the two kNN models applied on the training set was significant (P value = 0.0040). The differences in performance of the kNN model were most pronounced for prediction of short life expectancy. For the clinical parameters only model as applied on the training set, of the individuals with predicted life expectancy less than 2.5 years (n = 20), 13 survived longer than 3.5 years. When supplementing the clinical parameters with gene expression data, the model predicted 11 individuals to have life expectancy less than 2.5 years, and only 4 of them survived more than 3.5 years.

In order to further investigate which patient group would benefit more in terms of the improved survival prediction accuracy by using kNN modeling, another prediction model for patient group classification was performed. The grouping variable was modeled as a category variable, four clinical parameters and the expression levels of two genes were modeled as continuous variables. This model was used to estimate whether the use of gene variables could improve the prediction of classification for each patient group beyond that estimated using the available clinical parameters only (Fig 2). ROC curves for grouping variable prediction were estimated to show the sensitivity and the specificity of survival prediction. As compared to the prediction model that used only the clinical parameters, the addition of the expression levels of IGFBP3 and F3 increased the prediction performance in three groups, except in the PCa group with a slightly lower performance (Fig 2, Panel (A)). The Area Under the ROC Curve (AUC) value was increased in OD, MA and RA patient groups. Particularly for the two alive patient groups with much longer survival time, AUC value was increased from 0.627 to 0.690 and from 0.787 to 0.848, respectively (Fig 2, Panel (B)).

## Discussion

Prostate cancer is a disease that has an increasing incidence and prevalence but constant mortality. Diagnostic routines have reached the efficiency of finding both the aggressive prostate cancers and the slow-growing cancers that will not affect the patient in any serious manner. With limited resources for treatment, and with serious side effects of treatment complications in those patients predominantly with slow-growing tumors, better stratification tools are required so that only aggressive cancers are given required treatment [12]. This work evaluates the ability of two genes, IGFBP3 and F3, as analyzed in FFPE core needle biopsy tissue, to provide prognostic information about the expected survival time of a prostate cancer patient when combined with currently used clinical parameters. The genes were previously identified in a report from our laboratory, but were at that time evaluated using a different sample type (fresh frozen FNA cytological samples) [3,13]. FFPE samples are of higher relevance in current clinical practice.

When viewed as univariate parameters, the gene expression levels of IGFBP3 and F3 both had the ability to distinguish at least two of the four patient groups each. IGFBP3 was expressed to a lower extent in the MA and OD groups, and F3 was expressed most abundantly in the RA group, and had the lowest expression in the PCa group. The variation of the values within each group was however high, meaning that gene expression values alone will not be adequate as a prognostic tool in clinical practice, and only in combination with other variables a robust decision support can be provided.

The clinical parameters evaluated in this study largely followed anticipated patterns: The PCa group had higher Gleason score, log10 (PSA) and tumor stage than the OD group and the RA group. Clinical parameters alone had a clear ability to estimate survival time, but performance could be improved through addition of expression levels of the two genes. Since we also observed that F3 expression levels could provide an additional value to stratify the ‘baseline’ group (RA group) with the other three groups after IGFBP3 distinguished MA alive patients from deceased groups. The combination of IGFBP3 and F3 would provide better classification ability compared to using IGFBP3 only, which indicates a pattern recognition method that can handle non-linear relationships is more desirable such as kNN model.

In the original design of the groups, MA group was matched with respect to age and Gleason score for the combined PCa and OD groups. About 17% of the originally selected cases could not be retrieved and the drop-out cases have affected the original matching leading to Age being significantly different when comparing the MA group with the (PCa + OD) group (Table 2). Analysis of age with the MA group must therefore be handled carefully. That being said, the importance of age for stratifying the MA group from the OD group is interesting and might imply that age of patients plays a dominant role in overall survival prediction for those patients who have lower Gleason score, PSA or tumor stage. More data is required to confirm this finding in the future.

In order to verify the survival time prediction value of the gene signature in a new individual cohort with more obvious significance, we selected different patient groups with different characteristics such as the matched alive group having higher GS and elder age of patients compared to the randomly selected alive patient group, and the two types of deceased patient groups having much shorter overall survival time. Also we observed that the GS and age of patients were not perfectly matched between the MA patient group and the deceased patients groups (PCa and OD groups). When the MA group was compared to the PCa group, only age variable was not significantly different while there was a difference for GS, which means that the difference in expression levels of IGFBP3 might be affected by GS as well. Multiple linear regression analyses however confirmed that IGFBP3 and F3 were significantly contributing to stratification power. This indicates Gleason score and two genes (IGFBP3 and F3) would provide the largest two weights for predicting overall survival in prostate cancer patients using a combined model including genes and clinical parameters together.

When comparing the results of the present study to our previous report [3] the first observation was the difference in cohort characteristics. The present study was based on a cohort with earliest diagnosis date in 2004 with a wide spread of patient and tumor characteristics. In the previous report, a historic cohort with cases from 1986–2001 was evaluated, mainly with elderly men who had advanced cancer, very high PSA values and who were treated with hormone therapy alone. This means that the performance profile of all investigated parameters will be difficult to compare. The sample type (FFPE tissue in the present study, cytology samples in the previous report) also showed differences, where the average RNA quantity in the FFPE sample was smaller than in the cytology samples. This was a challenge for the analytical procedures to measure gene expression. Even though there were differences in design, the two independent studies shared one important feature: In both cases, IGFBP3 and F3 were related to survival time and estimates of survival were improved when gene expression information was added to currently used clinical parameters.

The third gene (VGLL3) discussed in the previous reports [3,9] was not included in the present study. The underlying reason for VGLL3 failure was insufficient quantity of VGLL3 mRNA leading to need for larger quantities of tissue to accurately determine gene expression [9]. The data for the present study was collected before the VGLL3 tissue quantity issue was confirmed. Protocols for tissue harvesting have been amended so that future cohorts will obtain valid measurements for VGLL3. The performance of the VGLL3 gene on FFPE tissue samples will be reported at a later stage. An alternative to measuring mRNA of VGLL3 would be to measure protein expression level of VGLL3 using immunohistochemistry or using FNA for sampling.

The kNN method was used to evaluate the survival prediction performance by comparing the average absolute prediction error. In this study, we did not perform any survival time correction for those patients who received treatment. Many of the actively treated patients did benefit from their treatments in terms of prolonged life. Under this condition, comparing survival time of untreated patients with treated patients becomes complicated and inflicts bias when investigating the pure effects from genes or any other clinical parameters on prognosis. The alternative would be to correct survival time based on estimated treatment effect, but it would be difficult to justify any technical solution for such a correction. Considering that the cohort contains biopsies from all registered prostate cancer deaths during 4 years within the Stockholm Gotland region (with approximately 2 million inhabitants), it would take long time to collect a cohort of sufficient size to distinguish between treatment options. Hence, analysis with actual survival time is the best option under current circumstances.

Overall survival is the real lifetime determined by a combination of the aggressiveness of prostate cancer and patient’s other conditions such as comorbidities. The patients in this cohort were diagnosed between 2004 and 2007, and the registry follow up was until the end of 2013. Using 5-year overall survival as end point was probably the best choice, because it is a clinically used divider between aggressive cancer that requires treatment and cancer that does not. Alternative surrogate end-points, such as biochemical recurrence, are convenient in the manner that a cohort can be collected in shorter time, but have the disadvantage that they are not perfectly representative of true overall survival, in particular not with respect to the influence of patient’s other conditions on overall survival. We have the privilege to work in an environment where tissue samples are archived in bio-banks and relevant clinical parameters are registered in regional and national databases, making it at all possible to use overall survival as the end-point.

With respect to the quality of cancer patients’ lives, the active surveillance program gets increasing attention for the management of prostate cancer treatments. A recent study reports that an active surveillance program could be recommended for up to two thirds of newly diagnosed prostate cancer patients when using published criteria, including a subset of the patients who were assigned for radical prostatectomy treatment [14]. From these criteria, the pathological parameters, such as Gleason grading and tumor cell percentage were reported to be critical parameters to assign patients into active surveillance program [15]. We report that by supplementing clinical parameters with the expression levels of IGFBP3 and F3, the ability to predict survival time is improved, which could be mainly derived from the better prediction accuracy for the alive patients: MA and RA groups (Fig 2). This implies the utilization of IGFBP3 and F3 combined with clinical parameters might provide improved stratification ability for those patients who are tending to assign to active surveillance program. The digitalized pathology image guided tissue sample taking methodology used in this study would also provide more precision for pathological diagnosis. Hence, with further follow-up of this cohort, or another cohort study with even longer survival time and longer than 10 years follow-up, the implication for patients who might benefit from active surveillance program would become evident in the future.

Seen together, gene expression of IGFBP3 and F3 in combination with clinical parameters such as Gleason score most probably has an important role to play in the stratification of newly diagnosed prostate cancer patients. The results reported in this study warrants initiation of additional studies to evaluate the use of gene expression as a complement to clinical parameters to improve prediction of prostate cancer patient prognosis. If studies with lager cohorts and survival follow up exceeding 10 years become available, survival prediction and treatment choice would be improved, in particular for patients who would be safely assigned to active surveillance.

## Supporting Information

S1 Table Multiple linear regression analyses. The expression levels of genes, delta Ct IGFBP3 and delta Ct F3, were fit in the multiple linear regression models with other parameters in order to investigate whether the association between ‘gene variable’ and ‘grouping variable’ was affected by the other clinical parameters: age, GS, Log (PSA) and clinical stage (S1 Table). MA group and RA group were separately integrated with PCa and OD groups to compose two major types of models: Type 1: MA, OD and PCa groups (A) for IGFBP3 and (B) for F3; Type 2, RA, OD and PCa groups (C) for IGFBP3 and (D) for F3.
(DOCX)

S2 Table Delta Ct values indicating the expression levels of IGFBP3 and F3. (DOCX)
