# Supplementary material for: Improving the Prediction of Prostate Cancer Overall Survival by Supplementing Readily Available Clinical Data with Gene Expression Levels of IGFBP3 and F3 in Formalin-Fixed Paraffin Embedded Core Needle Biopsy Material
Source: PLoS One. 2016 Jan 5;11(1):e0145545. doi: 10.1371/journal.pone.0145545 (PMC4701463; doi:10.1371/journal.pone.0145545)
Supplement: S1 Table — The expression levels of genes, delta Ct IGFBP3 and delta Ct F3, were fit in the multiple linear regression models with other parameters in order to investigate whether the association between ‘gene variable’ and ‘grouping variable’ was affected by the other clinical parameters: age, GS, Log (PSA) and clinical stage (S1 Table). MA group and RA group were separately integrated with PCa and OD groups to compose two major types of models: Type 1: MA, OD and PCa groups (A) for IGFBP3 and (B) for F3; Type 2, RA, OD and PCa groups (C) for IGFBP3 and (D) for F3. (DOCX) [file pone.0145545.s001.docx]

**S1 Table: Multiple linear regression analyses.**

| **Groups: MA, OD and PCa** | | |  |  |  | **Groups: RA, OD and PCa** | | |  |  |
| --- | --- | --- | --- | --- | --- | --- | --- | --- | --- | --- |
| **A. IGFBP3 associates with grouping variable and other four clinical variables** | | | | | | **C. IGFBP3 associates with grouping variable and other four clinical variables** | | | | |
| Model: IGFBP3 ~ (Group + Age + GS + Tumor Stage + logPSA)^a^ | | | | |  | Model: IGFBP3 ~ (Group + Age + GS + Tumor Stage + logPSA)^a^ | | | | |
| Term | Estimate | Std Error | t Ratio | Prob >\|t\| |  | Term | Estimate | Std Error | t Ratio | Prob >\|t\| |
| (Intercept) | 6.86 | 1.23 | 5.58 | <0.0001* |  | (Intercept) | 5.91 | 1.30 | 4.54 | <0.0001* |
| Group_OD (Ref: Group_MA) | -0.02 | 0.38 | -0.04 | 0.9647 |  | Group_OD (Ref: Group_RA) | 1.67 | 0.43 | 3.88 | 0.0002* |
| Group_PCa (Ref: Group_MA) | -0.88 | 0.40 | -2.18 | 0.0303* |  | Group_PCa (Ref: Group_RA) | 0.74 | 0.47 | 1.59 | 0.1148 |
| Age | 0.00 | 0.02 | -0.29 | 0.7751 |  | Age | -0.01 | 0.02 | -0.66 | 0.5127 |
| GS=7 (Ref: GS<=6) | -0.87 | 0.42 | -2.07 | 0.0397* |  | GS=7 (Ref: GS<=6) | -1.29 | 0.43 | -2.96 | 0.0036* |
| GS>=8 (Ref: GS<=6) | -1.26 | 0.48 | -2.61 | 0.0099* |  | GS>=8 (Ref: GS<=6) | -1.49 | 0.53 | -2.79 | 0.0058* |
| TumorStage_T2 (Ref: T=1) | -0.70 | 0.41 | -1.69 | 0.0931 |  | TumorStage_T2 (Ref: T=1) | -0.55 | 0.44 | -1.25 | 0.2142 |
| TumorStage_T3/T4 (Ref: T=1) | -0.87 | 0.46 | -1.90 | 0.0596 |  | TumorStage_ T3/T4 (Ref: T=1) | -0.60 | 0.51 | -1.17 | 0.2441 |
| logPSA | -0.35 | 0.30 | -1.14 | 0.2577 |  | logPSA | -0.38 | 0.32 | -1.18 | 0.2398 |
|  |  |  |  |  |  |  |  |  |  |  |
| R Square | 0.25 |  |  |  |  | R Square | 0.24 |  |  |  |
| Adjusted R Square | 0.22 |  |  |  |  | Adjusted R Square | 0.20 |  |  |  |
| Observations | 179 |  |  |  |  | Observations | 170 |  |  |  |
|  |  |  |  |  |  |  |  |  |  |  |
| **B. F3 associates with grouping variable and other four clinical variables** | | | | | | **D. F3 associates with grouping variable and other four clinical variables** | | | | |
| Model: F3 ~ (Group + Age + GS + Tumor Stage + logPSA)^a^ | | | | |  | Model: F3 ~ (Group + Age + GS + Tumor Stage + logPSA)^a^ | | | |  |
| Term | Estimate | Std Error | t Ratio | Prob >\|t\| |  | Term | Estimate | Std Error | t Ratio | Prob >\|t\| |
| (Intercept) | 2.21 | 1.90 | 1.16 | 0.2477 |  | (Intercept) | 3.94 | 1.87 | 2.10 | 0.0372* |
| Group_OD (Ref: Group_MA) | -0.17 | 0.58 | -0.30 | 0.7660 |  | Group_OD (Ref: Group_RA) | 0.95 | 0.62 | 1.54 | 0.1259 |
| Group_PCa (Ref: Group_MA) | 0.59 | 0.62 | 0.94 | 0.3476 |  | Group_PCa (Ref: Group_RA) | 1.59 | 0.67 | 2.37 | 0.0191* |
| Age | 0.01 | 0.03 | 0.35 | 0.7285 |  | Age | -0.03 | 0.03 | -0.94 | 0.3489 |
| GS=7 (Ref: GS<=6) | 0.89 | 0.65 | 1.37 | 0.1727 |  | GS=7 (Ref: GS<=6) | 0.33 | 0.63 | 0.53 | 0.5984 |
| GS>=8 (Ref: GS<=6) | 0.84 | 0.75 | 1.12 | 0.2643 |  | GS>=8 (Ref: GS<=6) | 0.65 | 0.77 | 0.84 | 0.3999 |
| TumorStage_T2 (Ref: T=1) | 0.02 | 0.64 | 0.03 | 0.9749 |  | TumorStage_T2 (Ref: T=1) | -0.05 | 0.64 | -0.08 | 0.9331 |
| TumorStage_ T3/T4 (Ref: T=1) | 0.80 | 0.71 | 1.13 | 0.2582 |  | TumorStage_ T3/T4 (Ref: T=1) | 0.89 | 0.74 | 1.22 | 0.2255 |
| logPSA | -0.86 | 0.47 | -1.83 | 0.0691 |  | logPSA | -0.82 | 0.47 | -1.77 | 0.0783 |
|  |  |  |  |  |  |  |  |  |  |  |
| R Square | 0.06 |  |  |  |  | R Square | 0.09 |  |  |  |
| Adjusted R Square | 0.01 |  |  |  |  | Adjusted R Square | 0.05 |  |  |  |
| Observations | 179 |  |  |  |  | Observations | 170 |  |  |  |

Abbreviations: Abbreviations: PCa: Death due to prostate cancer; OD: Death due to other causes; MA: Matched Alive more than 5 years; RA: Randomized Alive more than 5 years. If Prob >|t| is significant (<0.05), a star is marked.
