# Supplementary material for: Improving the Prediction of Prostate Cancer Overall Survival by Supplementing Readily Available Clinical Data with Gene Expression Levels of IGFBP3 and F3 in Formalin-Fixed Paraffin Embedded Core Needle Biopsy Material
Source: PLoS One. 2016 Jan 5;11(1):e0145545. doi: 10.1371/journal.pone.0145545 (PMC4701463; doi:10.1371/journal.pone.0145545)
Supplement: S2 Table — (DOCX) [file pone.0145545.s002.docx]

S2 Table. Delta Ct values indicating the expression levels of IGFBP3 and F3

| Delta Ct IGFBP3 | Delta Ct F3 | Delta Ct IGFBP3 | Delta Ct F3 | Delta Ct IGFBP3 | Delta Ct F3 | Delta Ct IGFBP3 | Delta Ct F3 |
| --- | --- | --- | --- | --- | --- | --- | --- |
| 3,6 | -0,1 | 5,1 | 5 | 3,7 | 2,7 | 5,8 | 1,6 |
| 1,8 | 2,4 | 9,3 | 0,8 | 10,5 | 3,8 | 2,6 | 5,2 |
| 4,3 | 5,9 | 4,9 | 1,2 | 3,4 | 8 | 7,5 | 8,2 |
| 5,4 | 1,8 | 4,3 | 5 | 2,7 | 4,5 | 4 | 1,5 |
| 6,9 | 2,8 | 3 | 2,5 | 3,9 | 3,1 | 2,2 | 6,4 |
| 8,3 | 5,7 | 1,1 | 0,9 | 1,8 | 12 | 3,4 | 1,9 |
| 3,6 | 10 | 2,9 | 1 | 1,9 | 1,6 | 7,6 | 5,1 |
| 3,5 | 11,1 | 6,5 | 0,6 | 0,7 | -3 | 2,2 | 0,2 |
| 6,5 | 1,7 | 4,9 | 3,4 | 5,4 | 2,5 | 4,1 | 0,9 |
| 2,2 | 2,2 | 4,3 | 1,2 | 2,8 | 2,3 | 3,6 | 10 |
| 10,1 | 4,1 | 5,5 | 2,6 | 1,1 | 0,1 | 3,1 | 3,3 |
| 4,4 | -0,1 | 2,5 | 9,2 | 4,7 | 1,1 | 4,4 | -1,4 |
| 1,6 | 0,4 | 3,7 | 1,7 | 1,9 | 4,2 | 5,2 | 2,5 |
| 3,9 | 7,7 | 4 | 0,7 | 3,1 | 11,2 | 4,5 | 3 |
| 4,4 | -1,4 | 2,6 | 3,3 | 3,9 | 1,1 | 1,9 | 0,2 |
| 4,9 | 12,1 | 6,2 | 1,6 | 0,9 | 10 | 8,2 | 7,2 |
| 3,8 | 2,5 | 2,1 | 1,3 | 8 | 4,4 | 2,5 | 0,8 |
| 5,2 | 2,5 | 4,5 | 1,1 | 6,1 | 3,9 | 2,4 | 0,7 |
| 4,5 | 3 | 6,3 | 3,3 | 3,9 | 0,7 | 1,6 | 2 |
| 3,8 | 1,8 | 3,1 | 3 | 1,9 | 6,7 | 2,6 | 1,2 |
| 4,1 | 1,1 | 4,1 | 1,7 | 5,9 | 3,3 | 4,9 | 1,5 |
| 1,5 | 2,9 | 10,1 | 10,8 | 5,7 | 2 | 3,7 | 0,4 |
| 1,2 | 3,5 | 3,9 | 1,2 | 4,8 | 1,8 | 6,3 | 3,3 |
| 2,4 | -1,9 | 5,4 | 2,6 | 4,1 | -0,1 | 3,9 | 1,2 |
| 4,5 | 1,9 | 6,3 | 2,3 | 3,8 | 2,8 | 1,9 | 1,1 |
| 5,5 | 10,6 | 6 | 1,7 | 0,9 | 4,8 | 6 | 1,7 |
| 8,2 | 7,2 | 4,5 | 4,8 | 4,9 | 1,3 | 5,5 | 0,8 |
| 5,7 | 0,9 | 1,3 | 3,7 | 2,6 | 2 | 0,8 | -0,2 |
| 2,3 | 9,3 | 3,7 | 3,4 | 5,1 | 0,9 | 5,8 | 2,4 |
| 2,6 | 7,1 | 3,8 | 1 | 5,5 | -0,6 | 1,7 | 1,5 |
| 9,9 | 9,9 | 2,1 | 0,9 | 5 | 2,4 | 4,7 | -0,2 |
| 4,5 | 3,2 | 3,7 | 1,1 | 4,4 | 3,4 | 6,7 | 0 |
| 3,2 | 5,7 | 2,7 | -0,1 | 5,4 | 0,1 | 2 | -0,5 |
| 6,7 | -0,4 | 3 | 1,7 | 2,6 | 3,9 | 5,1 | 0,7 |
| 4 | 1 | 5,5 | 0,8 | 7,6 | -1,6 | 2,1 | 0,9 |
| 3,5 | 0,8 | 2,6 | 3,4 | 10,4 | 3,3 | 1,7 | -0,4 |
| 2,3 | 0,3 | 8,2 | 0,2 | 3,7 | 3,6 | 0,9 | 1,4 |
| 9,4 | 9,4 | 2,8 | 1,4 | 3,8 | 1,4 | 1 | 0,4 |
| 6,3 | 3,5 | 4,6 | 3,3 | 3,5 | 4,3 | 1,9 | 0,6 |
| 8,8 | 2,7 | 8,9 | 2,3 | 5 | 2,4 | 0,6 | 1,4 |
| 9,8 | -2,9 | 1,1 | 0,9 | 1 | 4,3 | 10,5 | 2,7 |
| 5,1 | 1,7 | 5,8 | 3,5 | 5,1 | -0,1 | 3 | 0,4 |
| 3,2 | 4,8 | 2,6 | 0,4 | 0,2 | 3,3 | 3,5 | 2 |
| 7,7 | 0,8 | 4,4 | 5,1 | 3 | 2,4 | 2,2 | 0,6 |
| 3,8 | 0,4 | 2,4 | 0,3 | 3,2 | -1,5 | 2 | 0,2 |
| 2,6 | -0,3 | 6,6 | 4,2 | 6 | 0,5 | 4,3 | 2,1 |
| 3 | 2,2 | 5,8 | 2,4 | 2 | 1,5 | 2,8 | 1 |
| 4,8 | 8,2 | 2,4 | 2,2 | 5,3 | 0,5 | 2,3 | 1,5 |
| 5,8 | 5,5 | 1,7 | 2 | 3,5 | 0,6 | 2,6 | 0,2 |
| 3,9 | 7 | 5,1 | -1 | 0,3 | -1,1 | 2,4 | 0,9 |
| 4,3 | 2,3 | 4,3 | -0,9 | 10,3 | 2 | 3,1 | 2,6 |
| 4,9 | 1,5 | 3 | 2,6 | 6,2 | 1,8 | 4,3 | 0,4 |
| 2,6 | 3,6 | 1,7 | 7,9 | 7,5 | -0,1 | 3,4 | 3 |
| 3 | 0,1 | 4,5 | 6,4 | 5 | 2,2 | 3,1 | 2,5 |
| 3,5 | 6,7 | 2,5 | 2,6 | 3,7 | 1,2 | 2,6 | 2,1 |
| 2,9 | -1 | 3 | 1,8 | 3,2 | 4 | 0,5 | 4,8 |
| 2,6 | 1,1 | 4 | 1,2 | 5,4 | 11,5 | 6,2 | 1,8 |
| 4,5 | -0,2 | 4,7 | -0,2 | 4,8 | 0,7 | 4,8 | 0,7 |
| 11,2 | 1,6 | 3,2 | 2,1 | 3,3 | 4,1 | 9,8 | 9,8 |
| 4,8 | 0,6 | 5,6 | 8,5 | 2,5 | -1,6 | 3,4 | 1,9 |
|  |  |  |  |  |  | 7,6 | 5,1 |
